# ENO1 Overexpression in Pancreatic Cancer Patients and Its Clinical and Diagnostic Significance

**DOI:** 10.1155/2018/3842198

**Published:** 2018-02-01

**Authors:** Hang Yin, Lei Wang, Hai-Lin Liu

**Affiliations:** Department of Gastroenterology, The Ninth People's Hospital, Shanghai Jiao Tong University School of Medicine, Shanghai, China

## Abstract

We investigated in this study the expression of ENO1 in tissues and plasma of PDAC patients to evaluate its clinicopathological and diagnostic significance. ENO1 protein expression was detected in tissue microarray of human PDAC and adjacent noncancer tissues. Electrochemiluminescence immunoassay and amplified luminescent proximity homogeneous assay (AlphaLISA) were performed to measure CA19-9 and ENO1 concentration in plasma from PDAC patients and healthy controls. We demonstrated that ENO1 overexpression is positively correlated with clinical stage, lymph node metastasis, and poor prognosis of PDAC; ENO1 may function as a hopeful candidate diagnostic marker in combination with CA19-9 in PDAC diagnosis.

## 1. Background

The incidence of pancreatic cancer (PC) has been increasing rapidly in recent years, and it is currently the fourth leading cause of cancer deaths for both men and women [1, 2]. Most of PCs are pancreatic ductal adenocarcinomas (PDAC), which is characterized by a late presentation. The five-year survival rate of PDAC is less than 5% for all stages. This poor prognosis is related to the advanced disease stage at the time of diagnosis, which could be alleviated by its early detection [3, 4].

CA19-9 is a traditional tumor marker widely used for diagnosis of PC; however, the sensitivity and specificity of CA19-9 to diagnose PC is not satisfactory. CA19-9 belongs to blood antigens of the Lewis group, and individuals with Lewis-negative phenotype are unable to synthetize CA19-9. Approximately 5–10% of the population are Lewis negative, which may lead to false-negative results of CA199 in PC patients [5]. In addition, CA19-9 is a gastrointestinal tumor-associated antigen associated with gastric cancer, colorectal cancer, ampullary carcinoma, hepatocellular carcinoma, and extrahepatic bile duct cancer. Slight increase of CA19-9 is also observed in some benign lesions accompanied with bile duct obstruction such as hepatitis and acute pancreatitis [6, 7]. Therefore, it is urgent to seek new tumor marker to improve PC diagnosis.

Enolase, also known as pyruvate dehydrogenase, is a key glycolytic enzyme that catalyzes the dehydration of 2-phospho-d-glycerate to phosphoenolpyruvate. It has been shown that enolase 1 (ENO1) may play an important role in tumorigenesis, cancer invasion, and metastasis [8–12]. In our previous study, we showed a significant up-regulation of ENO1 in rat models of 7,12-dimethylbenzanthracene (DMBA)-induced pancreatic intraepithelial neoplasia (PanIN) and PC using proteomics tools [13]. Some other studies also confirmed that ENO1 was up-regulated at the mRNA and/or protein levels in PDAC cell lines, and ENO1 IgG antibody could be detected in the peripheral blood of PDAC patients [14–17]. These results suggest that ENO1 may have the potential value to be a novel tumor marker for PC diagnosis.

In the present study, we investigated the differential expression of ENO1 in human PDAC and adjacent non-cancer tissues, as well as peripheral blood of PDAC patients and healthy controls, to explore its clinicopathological significance, and diagnostic value as tumor marker single or combined with CA19-9.

## 2. Material and Methods

### 2.1. Specimens

A tissue microarray (Shanghai Outdo Biotech Co. Ltd., OD-CT-DgPan03-002, 19 males and 12 females) containing 31 pairs of human PDAC and adjacent noncancer tissues was used for IHC staining. All cases were staged according to the seventh edition of the pancreatic cancer TNM staging system of the American Association of Cancer (AJCC) in 2010 [18] including 4 stage I, 4 stage II, and 23 stage III. The samples were formalin-fixed and embedded in paraffin.

Blood samples from 73 PC patients without jaundice were collected from October 2012 to October 2014 and followed up until October 2015 in the Department of Gastroenterology, Shanghai Ninth People's Hospital, Shanghai Jiao Tong University School of Medicine. The cases were comprised of 43 males and 30 females with an average age of 68.3 ± 11.5 years. All cases were classified as PDAC with pathologic examination by operation, endoscopic ultrasonography-guided fine-needle aspiration (EUS FNA) or endoscopic retrograde cholangiopancreatography (ERCP). No chemotherapy, radiotherapy, or surgery was performed to the patients when the samples were collected. Of the patients, the TNM classified 11, 19, 27, and 16 patients into stages I, II, III, and IV, respectively. Fifty healthy cases without digestive system disease or tumor history were enrolled as normal control (NC) during the same period, in which 27 cases were male and 23 were female with an average age of 63.6 ± 6.96. There was no significant difference in the gender and age between the PDAC and NC group (*P* > 0.05).

Blood samples were collected in vacuum blood vessels containing EDTA anticoagulant, centrifuged with 3500 rpm for 15 min after standing at room temperature for 30 min. The plasma samples were stored at −80°C before use. This study was approved by the Ethics Committee of Shanghai Ninth People's Hospital, Shanghai Jiao Tong University School of Medicine. The use of blood samples was informed and authorized by all patients and healthy controls.

### 2.2. Immunohistochemistry (IHC) Staining

The tissue microarray was deparaffinized sequentially in xylene and alcohol and washed three times in phosphate buffered saline (PBS). The endogenous peroxidase activity was blocked with 3% hydrogen peroxide in PBS for 10 min, and the sections were then incubated with 0.05% trypsin for 30 min at 37°C. After three washes with PBS, nonspecific binding was blocked with protein block for 30 min at 37°C. The sections were then incubated overnight at 4°C with an anti-ENO1 antibody (WH0002023M1, Sigma-Aldrich, St. Louis, MO, USA) at a dilution of 1 : 400. DAB (1 : 50) was utilized to detect ENO1 protein with the deposition of a brown reaction product in the nuclei and cytoplasm of the positive cells. Sections incubated with PBS instead of the anti-ENO1 antibody served as control. IHC staining was graded by blinded observers using two semiquantitative measurements: staining intensity (0–4) and percentage of cells stained (0 = no staining, 1 = less than 25%, 2 = 25%–50%, 3 = 50%–75%, and 4 = 75%–100%). A combined IHC score was calculated as the product of staining intensity and percentage of stained cells.

### 2.3. Quantitative Determination of CA19-9 and ENO1 in Plasma

Plasma concentration of CA19-9 was measured using the quantitative electrochemiluminescence immunoassay kit on the Roche e601 system (Roche Diagnostics, Mennheim, Germany). All procedures were performed according to the manufacturer's instructions.

Plasma concentration of ENO1 was determined by amplified luminescent proximity homogeneous assay (AlphaLISA) method as described previously [19, 20]. Anti-ENO1 monoclonal antibodies (WH0002023M1, SAB1403772) and polyclonal antibodies (AV34376, E2659) were obtained from Sigma-Aldrich (St. Louis, MO, USA). AlphaLISA reagents (PerkinElmer, Waltham, MA, USA) consisted of AlphaLISA unconjugated acceptor beads (6772001), streptavidin donor beads (6760002S), and AlphaLISA immunoassay buffer (AL000C). AlphaLISA assays were performed in 96-well Optiplates and read in an EnSpire^TM^ Multilabel Plate reader (PerkinElmer, Waltham, MA, USA).

### 2.4. Statistical Analysis

Data were analyzed using IBM SPSS statistic 19 (IBM, Armonk, NY, USA). The chi-square tests were used to determine the ENO1 expression differences between PDAC and adjacent noncancer tissues. The Wilcoxon rank-sum test and bilateral test were used in pairwise comparison of ENO1 and CA199 plasma levels between the groups. The correlation of plasma ENO1 level and the patient characteristics in the PDAC group was evaluated by Spearman's correlation analysis. To explore the correlation between ENO1 expression and prognosis of PDAC, the median of plasma ENO1 concentration in peripheral blood was used as the cutoff, and survival data were analyzed using the Kaplan-Meier method with a log-rank test for comparison. A logistic regression analysis was utilized to draw a receiver operating characteristic (ROC) curve. The area under the curve (AUC) was calculated to compare the performance of different biomarkers as a diagnostic test. *P* < 0.05 was considered to indicate a statistically significant difference.

## 3. Results

### 3.1. ENO1 Was Up-Regulated in Tissues and Plasma of PDAC Patients

**Figure 1:**
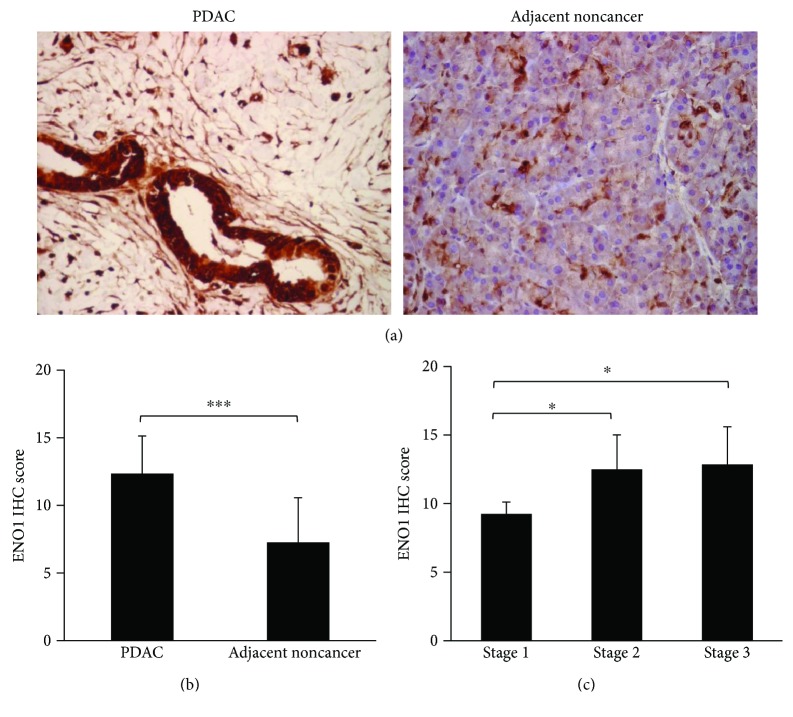
The expression of ENO1 in human PDAC and adjacent noncancer tissues. (a) Representative images showing IHC staining of ENO1 protein in human PDAC and adjacent noncancer tissues. Brown staining indicated ENO1 expression. Immunoreactivity level of ENO1 was higher in the nucleus and cytoplasm but weaker on the membrane. (b) Bar plots represented IHC score for ENO1 expression in human PDAC and adjacent noncancer tissues (*n* = 31). (c) Bar plots represented IHC score for ENO1 expression in human PDAC tissues of stage I (*n* = 4), stage II (*n* = 4), and stage III (*n* = 23). ^∗^*P* < 0.05, ^∗∗∗^*P* < 0.001.

**Figure 2:**
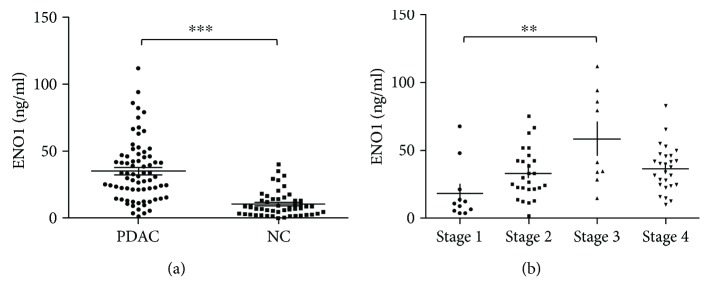
The level of ENO1 in plasma of PDAC patients, corresponding to clinical stages and prognosis. (a) Scatter plot demonstrated the ENO1 concentrations in plasma of PDAC patients (*n* = 73) and normal controls (*n* = 50). ENO1 was detected with amplified luminescent proximity homogeneous assay (AlphaLISA) method. (b) Scatter plot represented the ENO1 concentrations in plasma of PDAC patients with different clinical stages (stage I: *n* = 11; stage II: *n* = 19; stage III: *n* = 27; stage IV: *n* = 16). ^∗∗^*P* < 0.01, ^∗∗∗^*P* < 0.001.

The expression of ENO1 was compared in PDAC and adjacent noncancer tissues in PDAC tissue microarray. IHC staining showed that the expression level of ENO1 was higher in the nucleus and cytoplasm, but weaker in the membrane (Figure 1(a)). The expression of ENO1 was significantly increased in human PDAC tissues (*P* < 0.001), with an ENO1 IHC score of 12.34 ± 2.79 in human PDAC tissues in comparison with 7.26 ± 3.31 in adjacent noncancer tissues (Figure 1(b)). Up-regulation of ENO1 concentration was also found in the plasma of 73 PDAC patients as compared to that of the normal controls. The ENO1 concentration in the PDAC group was 33.08 ± 22.87 ng/ml, which was significantly higher than its concentration of 10.40 ± 9.41 ng/ml in normal control group (*P* < 0.001) (Figure 2(a)).

### 3.2. The ENO1 Expression of Tissues and Plasma Was Related to PDAC Development

The results indicated that the ENO1 levels in tissues and peripheral blood of PDAC patients were related to the regional development of the primary tumor. In different stages of PDAC tissues in tissue microarray samples, the ENO1 IHC score was increased from 9.25 ± 0.87 (stage I), 12.5 ± 2.52 (stage II), to 12.85 ± 2.76 (stage III) (*P* < 0.05) (Figure 1(c)). The plasma ENO1 levels at different PDAC stages were detected as follows: 19.03 ± 3.66 ng/ml of stage I, 30.26 ± 4.18 ng/ml of stage II, 61.74 ± 15.86 ng/ml of stage III, and 31.72 ± 5.35 ng/ml of stage IV. The ENO1 concentration of peripheral blood had a positive association with PDAC stage, as the plasma ENO1 was increased in stage II, stage III, and stage IV compared with stage I, although only significant difference was observed between stage I and stage III (*P* < 0.01) (Figure 2(b)). Further analysis showed the level of ENO1 in lymph node metastasis group (9.23 ± 4.68 ng/ml) was higher than that in no lymph node metastasis group (6.51 ± 4.69 ng/ml) (*P* < 0.01). There were no correlations between the ENO1 level and the site of PDAC, distant metastasis such as liver metastasis, the patients' age and sex, and diabetes.

### 3.3. Plasma ENO1 Level Was Associated with the Prognosis of PDAC Patients

**Figure 3:**
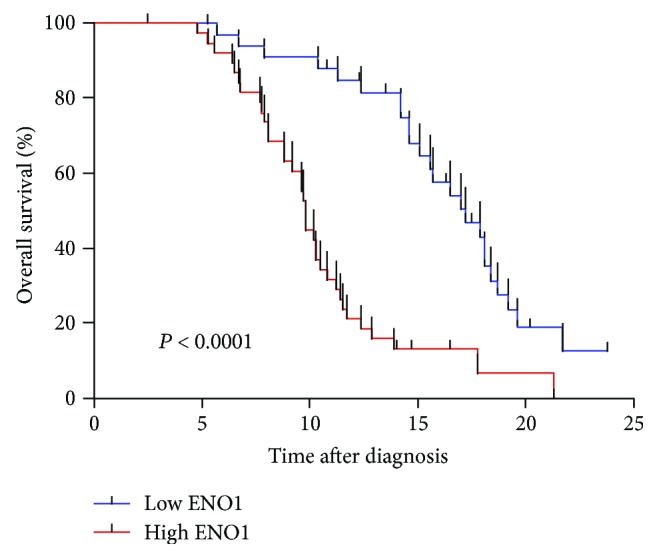
Overall survival curve with median value as cutoff for ENO1 represented the association between the plasma ENO1 level and prognosis of PDAC patients.

The results showed that the elevated plasma ENO1 level was related to the poor prognosis of PDAC patients. When using the median value of 27.8 ng/ml as cutoff, the median survival time of PDAC patients with higher ENO1 level (>27.8 ng/ml) was lesser than that of PDAC patients with lower ENO1 level (≤27.8 ng/ml) significantly (*P* < 0.0001) (Figure 3). The median survival time of PDAC patients was 18.66 ± 2.43 months (95% confidence interval (CI), 13.91–23.42 months) with lower ENO1 concentration (≤27.8 ng/ml); in contrast, the median survival time was decreased to 15.62 ± 2.44 months (95% CI 10.83–20.41 months) with higher ENO1 level (>27.8 ng/ml).

### 3.4. Diagnostic Value of ENO1 Single or Combined with CA19-9 for PDAC

**Figure 4:**
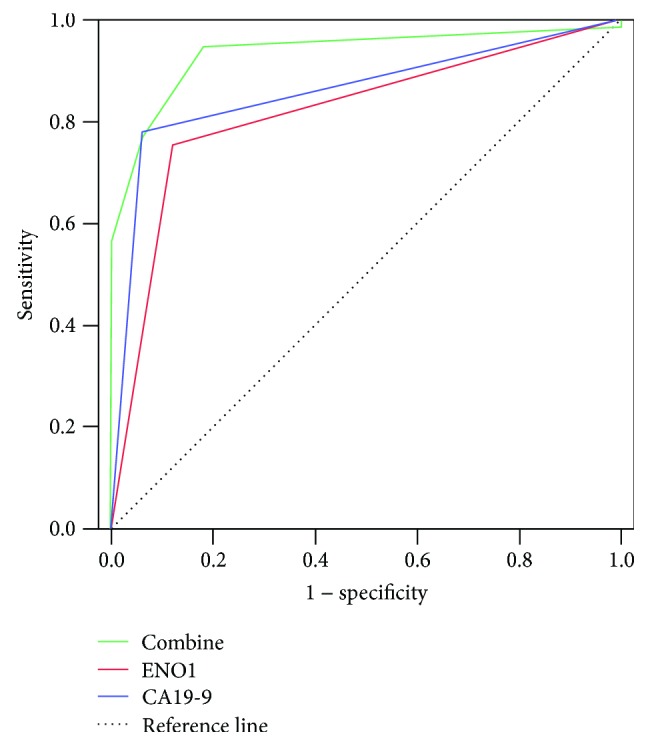
Diagnostic value of ENO1 single or combined with CA19-9 for PDAC. ROC curves of CA19-9, ENO1, and a combination of both were shown with the median value of ENO1 used as cutoff for diagnosing PDAC. *P* < 0.001.

The ROC curve was therefore generated to study the diagnostic value of ENO1 single or combined with CA19-9 for PDAC. The area under the curve (AUC) of CA19-9 was 0.869 (95% CI 0.791–0.929; *P* < 0.001). When the CA19-9 concentration of 37 U/ml was used as cutoff for distinguishing PDAC, the sensitivity and specificity of diagnosing PDAC were 78.1% and 94.0%, respectively. The AUC of ENO1 was 0.817 (95% CI 0.738–0.895; *P* < 0.001). When the median ENO1 concentration of 27.8 ng/ml was used as cutoff for diagnosing PDAC, the sensitivity and specificity of diagnosis were 75.8% and 88.2%, respectively. The AUC of the combination of CA19-9 and ENO1 was 0.935 (95% CI 0.889–0.980; *P* < 0.001), with the sensitivity and specificity of the two-marker panel improved to 94.5% and 82%, respectively (Figure 4).

Furthermore, of all 73 PDAC patients, the plasma CA19-9 levels were in the normal range in 15 PDAC patients. Of these 15 patients, 10 were identified with a plasma level of ENO1 higher than the proposed reference value of ≤12.88 ng/ml.

## 4. Discussion

Glycolysis is enhanced in most tumor cells which is named Warburg effect [21], while more than 50% of the energy is generated by glycolysis instead of the tricarboxylic acid cycle even in the presence of oxygen. Tumor cells can achieve rapid growth with a higher efficiency of glucose absorption and biological macromolecule synthesis by reprogramming metabolic procedure to enhance glycolysis pathway [22]. ENO1, also called alpha-enolase, is a key glycolytic enzyme catalyzing the conversion of 2-phospho-d-glycerate to phosphoenolpyruvate [9, 16]. It is known that genes in the glycolysis pathway have been found to be overexpressed in a set of cancers [23, 24]. As a metabolic enzyme in glycolysis pathway, significantly increased ENO1 expression was confirmed in the human PDAC tissues and plasma in this study. Other studies also showed that ENO1 expression was up-regulated at the mRNA and/or protein level in PDAC cell lines and animal model tissues [13–15].

Recent researches have shown that ENO1 plays an important role in several biological and pathophysiological processes [15, 16, 25], such as tumorigenesis, cancer invasion, and metastasis [26, 27]. ENO1 is also a potential target for immunotherapy, for it was reported that mouse antihuman ENO1 monoclonal antibodies inhibited the invasiveness of human PDAC cells [15]. In most cases, ENO1 is a cytoplasmic protein, but it can also be expressed on the cell membrane or in the form of a nuclear DNA binding protein, suggesting that ENO1 is a multifunctional enzyme. In the cytoplasm, ENO1 maintains the ATP level of the cells by regulating cell's reproduction and guarantees the survival of cells and execution of their physiological functions. In addition, ENO1 located in the cytoplasm may be associated with the cytoskeleton system and other metabolic enzymes to facilitate tumor cell movement. ENO1 localized on the cell membrane can activate the plasminogen system as a fibrin soluble plasminogen receptor, and the plasminogen system can promote the invasion and metastasis of tumor cells through participation of basement membrane and extracellular matrix remodeling [14]. Furthermore, a nuclear DNA binding protein named c-myc promoter binding protein-1 (MBP-1) is also encoded by the ENO1 gene, which is mainly located in the nucleus, binding with c-myc P2 promoter to negatively regulate c-myc expression and inhibit tumor growth [28].

Therefore, it should be reasonable that ENO1 has potential value to be a tumor marker for PDAC diagnosis. Although ENO1 was considered to be a potential tumor marker using proteomics in cell lines and tissues [29–32], few studies were carried out in the peripheral blood of PDAC patients. ENO1 can be discharged into the peripheral blood by necrosis and turnover of tumor cells or nonclassical secretory pathway such as exosome pathway. Prior studies proved that it was feasible to detect ENO1 in the body fluid [33], but we found it was difficult to detect ENO1 with the current commercial ENO1 enzyme-linked immunosorbent adsorption (ELISA) kit because its concentration in the peripheral blood is very low. Therefore, we selected the AlphaLISA method to detect ENO1 in the peripheral blood of PDAC patients. AlphaLISA technology is a bead-based method relying on the interaction between donor beads and acceptor beads. When the antibody-antigen reaction causes donor beads and acceptor beads to approach each other, the laser excites the cascade reaction, resulting in a greatly amplified signal [19, 20]. AlphaLISA method significantly improves the sensitivity of detection with obvious advantages and can be used as a powerful assay method for the development of ENO1 detection in the peripheral blood.

Our results showed that the ENO1 level was positively correlated with tumor progression in the peripheral blood of PDAC patients as well as in PDAC tissues, which indicated that ENO1 concentration may be associated with local tumor and vascular invasion. After reaching a peak at stage III, ENO1 plasma expression at stage IV cancer appeared to be lower than that at stage III cancer although the difference was not significant. This may be associated with the up-regulation of ENO1 which started at the very earliest neoplastic stage of PDAC [13], increased with the developing of PDAC, and decreased in the peripheral blood by necrosis and turnover of primary tumor cells at the latest stage. Stage IV cancer was not included in our tissue analysis because stage IV patients were mostly unable to receive surgery to collect tumor tissues. Furthermore, the ENO1 level was closely associated with the lymph node metastasis and the prognosis of PDAC. Patients with higher ENO1 concentration had significantly shorter survival time than those with lower ENO1 concentration, which indicated PDAC patients with higher ENO1 concentration should be revisited at shorter intervals to detect recurrence and metastasis. Our results were consistent with some additional studies that the ENO1 level was positively correlated with high degree of malignancy and poor prognosis [34–36].

Our results showed that ENO1 can be used as a hopeful diagnostic indicator for PDAC, and the combination of ENO1 and CA19-9 could improve diagnostic accuracy. When 27.8 ng/ml of ENO1 was used as cutoff for diagnosing PC, the sensitivity and specificity of diagnosis were 75.8% and 88.2%, respectively, and improved to 94.5% and 82% in combination with CA19-9, which was better than the sensitivity of CA19-9 (70–84.9%) [5]. It was noted that the sensitivity and specificity of ENO1 were almost close to CA19-9, and the sensitivity of ENO1 combined with CA19-9 was improved in comparison with either ENO1 or CA19-9. Furthermore, we found that the plasma concentrations of ENO1 in 10 of 15 PC patients with normal CA19-9 level were beyond the proposed reference value of ≤12.88 ng/ml, For PDAC patients of Lewis-negative phenotype with normal CA19-9 level, it necessitates caution when high level of ENO1 is detected.

As we included healthy participants as control, the specificity of ENO1 seems good in our study, but it is not specific enough to distinguish PDAC from other tumors. It has been shown that in hepatitis B virus-related hepatocellular carcinoma patients, ENO1 expression was enhanced in tumor tissue especially for poorly differentiated hepatocellular carcinoma and closely related to tumor size and vascular involvement [34]. The overexpression of ENO1 was associated with clinical stage and recurrence of non-small-cell lung cancer [30]. In head and neck cancer, patients with elevated expression of ENO1 suffered from worse clinical outcome including shorter overall and progression-free survival than those with lower expression [35]. Increased expression of ENO1 mRNA level was positively related with breast cancer size and lymph node metastasis but negatively related to disease-free interval [36]. ENO1 was highly expressed in the tumor tissues of patients with cholangiocarcinoma, which was positively correlated with peritoneal lymph node metastasis and prognosis [37]. Therefore, one important solution should be explored in future research is how to combine ENO1 with CT or MRI to improve diagnostic specificity and localization. As a vast majority of patients being screened for PDAC are patients who have chronic pancreatitis, to be a good diagnostic tool for PDAC, it is critical to know if ENO1 is present in patients with chronic pancreatitis. Although previous study showed no up-regulated expression of ENO1 mRNA in chronic pancreatitis tissues, similar to normal pancreas tissues [14], in the future study, we will additionally evaluate ENO1 levels of plasma as a distinguishing diagnosis marker in patients with chronic pancreatitis and PDAC.

## 5. Conclusion

The up-regulated level of ENO1 was positively correlated with disease progression and prognosis of PDAC. ENO1 may function as a hopeful candidate diagnostic marker in combination with CA19-9 in PDAC diagnosis.

## Abbreviations

AlphaLISA: Amplified luminescent proximity homogeneous assay
DMBA: 7,12-Dimethylbenzanthracene
ENO1: Enolase 1
ERCP: Endoscopic retrograde cholangiopancreatography
EUS FNA: Endoscopic ultrasonography-guided fine-needle aspiration
IHC: Immunohistochemical
MBP-1: c-myc promoter binding protein 1
PanIN: Pancreatic intraepithelial neoplasia
PBS: Phosphate-buffered saline
PC: Pancreatic cancer
PDAC: Pancreatic ductal adenocarcinoma.
